# Simultaneous Pancreas-Kidney Transplantation Versus Living Donor Kidney Transplantation Alone: an Outcome-Driven Choice?

**DOI:** 10.1007/s11892-018-1039-8

**Published:** 2018-07-20

**Authors:** Vishnu Swaroop Venkatanarasimhamoorthy, Adam D. Barlow

**Affiliations:** 1 Specialty Doctor in HPB and Transplant Surgery, St James’s University Hospital, Leeds, UK; 2 Consultant Transplant Surgeon, St James’s University Hospital, Beckett Street, Leeds, LS9 7TF UK

**Keywords:** Simultaneous pancreas-kidney transplantation, Living donor kidney transplantation, Transplant outcomes, Type 1 diabetes mellitus, Chronic kidney disease

## Abstract

**Purpose of Review:**

The choice of optimum transplant in a patient with type 1 diabetes mellitus (T1DM) and chronic kidney disease stage V (CKD V) is not clear. The purpose of this review was to investigate this in more detail—in particular the choice between a simultaneous pancreas-kidney transplantation (SPKT) and living donor kidney transplantation (LDKT), including recent evidence, to aid clinicians and their patients in making an informed choice in their care.

**Recent Findings:**

Analyses of large databases have recently shown SPKT to have better survival rates than a LDKT in the long-term, despite an early increase in morbidity and mortality in SPKT recipients. This survival advantage has only been shown in those SPKT recipients with a functioning pancreas and not those who had early pancreas graft loss.

**Summary:**

The choice of SPKT or LDKT should not be based on patient and graft survival outcomes alone. Individual patient circumstances, preferences, and comorbidities, among other factors should form an important part of the decision-making process. In general, an SPKT should be considered in those patients not on dialysis and LDKT in those nearing or already on dialysis.

## Introduction

Patients with T1DM and CKD V currently have the following transplant options—a deceased donor kidney transplant (DDKT), LDKT, or SPKT. That kidney transplantation offers significant survival benefit over remaining on dialysis is now well established [1]. Similarly, LDKT offers significant advantage over DDKT in terms of graft and patient survival [2, 3]. When it comes to comparing SPKT with LDKT, evidence is less convincing of the advantage of one over the other. There are no randomized controlled trials for any form of transplantation in this cohort of patients. Large registry studies have shown contradictory results between these two modalities of transplantation. This review aims to examine the evidence for these two modalities of transplant in further detail, to aid clinicians in making an informed choice of the most appropriate mode of transplant with their individual patients.

### SPKT Versus LDKT—Graft and Patient Survival Outcomes

Pancreas transplant outcomes have improved in recent times due to advanced surgical techniques [4] and better immunosuppression [5]. However, early mortality (within 90 days) is still significantly higher in SPKT than in LDKT or DDKT. This is due to the more complex procedure and the complications associated with it [6, 7]. In spite of this, studies have shown long-term survival benefits with SPKT compared to DDKT [8]. With regard to SPKT versus LDKT, the evidence is less clear. Morath et al. showed that long-term survival was better in SPKT than LDKT during years 10–18 after transplant [9] This survival benefit was because of less cardiovascular death and was noted specifically in those recipients with a functional pancreas at 10 years, indicating the benefit of long-term glycemic control in SPKT. In contrast, Young et al. demonstrated in a large OPTN/UNOS analysis that LDKT was associated with lower risk of death and graft loss [10]. This could have been due to the shorter follow-up in this study of 72 months, which may have introduced a bias against SPKT due to its higher early mortality risk as compared to LDKT. A recent analysis of the UK registry showed that there was no overall difference in patient survival in the two groups. However, those SPKT recipients with a functioning pancreas graft at 90 days had significantly better patient survival and similar kidney graft survival to LDKT recipients [11••]. It also demonstrated that LDKT was an independent predictor of improved kidney graft survival compared to SPKT. Similarly, other recent studies have highlighted the importance of early pancreas allograft survival to long-term outcomes. In a study of SPKT wait-listed patients, Weiss et al. showed that those patients who underwent surgery and had a functional pancreas at 12 months, had significantly better survival outcome over the following 7 years than those who had lost the pancreas early but still had a functioning kidney. This advantage was seen even over the group that received a LDKT. The group that suffered early pancreas loss showed a survival rate mirroring that of DDKT [12]. This is not surprising as loss of pancreas essentially leaves the SPKT recipient with a DDKT, outcomes of which are inferior to both SPKT and LDKT. The main reason for this lack of overall benefit seems to be due to the detrimental effects of pancreas graft loss on patient survival as well as kidney graft survival [11••]. A large retrospective analysis by Norman et al. showed that those SPKT recipients who had early pancreatic graft loss within 90 days had a 70% higher risk of kidney graft failure after 3 years and more than double the risk of death [13]. Another large single center study from Minnesota also showed similar results when the pancreas graft was lost within 90 days in SPKT recipients [14]. Therefore the focus in pancreas transplantation should be to improve pancreas graft outcomes as much as possible. To this effect, all efforts should be directed towards improving pancreas donor selection, pancreas assessment, organ preservation [4, 12], reduction of cold ischemia, and optimal management of complications after surgery. It must be noted that there is a high risk of selection bias in these observational studies, as access to the waiting list is hampered for the diabetes patients. This is mainly due to the fact that majority of the guidelines recommend strict screening criteria, especially for cardiovascular disease, in these patients [15]. Therefore, the above results are applicable in this group of type 1 diabetics, who passed these strict selection criteria and were eligible for an SPKT. Another bias to consider in these studies would be the referral bias in these highly specialized centers. SPKT is generally performed in high volume centers, and this affects the generalizability of outcomes from these centers. Also, the healthiest of these patients would be allocated to receive an SPKT, from the highest quality donors [16] and more often get a pre-emptive transplant [17, 18••].

In summary, these results indicate that patients with T1DM derive greater benefits over time from SPKT, as patient survival curves cross at the 5-year point in favor of SPKT. Therefore, the addition of a pancreas transplant in addition to a kidney transplant alone, confers long-term survival benefit in these patients, mainly due to euglycemia and reduced cardiovascular death. However, for those patients who have a living kidney donor available, LDKT is as good an option as SPKT, with a future PAK option in the short-term.

### Secondary Complications of Diabetes

Few studies have looked into the benefits of a pancreas transplant upon the secondary complications of diabetes including neuropathy [19], retinopathy [20] and nephropathy [21]. Most of these studies are small, single-centered without adequate controls or powered to be conclusive. A recent review by Boggi et al. looked at the impact of pancreas transplantation on secondary complications of diabetes. This review found that there is now growing data to show that a successful pancreas transplant may slow the progression, stabilize, and even favor the regression of some of these complications [22].

### Pre-Emptive Transplants

Increased mortality risk while awaiting transplant is inherent to those patients who are already on dialysis [23, 24]. Time spent on dialysis remains one of the strongest factors associated with poor kidney graft outcomes in these patients [24, 25]. Accumulation of dialysis time while awaiting either an SPKT or a LDKT is associated with reduced post-transplant survival [26]. However, a large retrospective analysis looking at a 7-year survival of preemptively transplanted LDKT versus SPKT with up to 2 years of dialysis time showed no difference in survival rates. This study also showed that pre-emptive SPKT provided comparable survival to LDKT with or without subsequent PAK [27].

### Patient Preference

Based on these findings, there is no clear advantage of one modality of transplant over the other. Therefore, individual patient preferences and circumstances must be taken into account before making a choice of transplant. Those patients with brittle diabetes and who are more troubled by hypoglycemic unawareness may benefit more from the euglycemic effect of a pancreas transplant and hence considered for an SPKT. Conversely, those patients for whom dialysis has a significant detrimental effect on their quality of life may be best served by the quicker cessation of dialysis offered by a LDKT. With regard to quality of life (QoL) after transplantation, there are only a few studies comparing SPKT to LDKT. A study by Sureshkumar et al. compared QoL in patients with T1DM who received SPKT, LDKT, and DDKT or were still on the waiting list. They reported an improvement only in diabetes-related QoL after SPKT but not in general QoL. But all modalities of transplantation showed a definite improvement in QoL over remaining on waiting list [28]. Similarly, a Spanish study showed improvement in QoL after SPKT compared those still on renal replacement therapy [29]. Ziaja et al. showed an improvement in QoL after SPKT compared to kidney transplant alone [30]. However, a study by Smith et al. reported an improvement in QoL in only half of SPKT recipients and a decrease in QoL in a third [31]. Past psychiatric disorder was a key factor in these patients. It is therefore important to educate patients before transplant regarding goals and treatment expectations. More studies are needed to look into this important aspect post transplant in this group of patients.

### Individual Patient Comorbidities

Traditionally, an age less than 50 years and BMI under 30 have been applied as criteria for selection of recipients by pancreas transplant centers. Advances in medical therapy and improved surgical outcomes in pancreas transplant mean that this age barrier is no longer applicable. Studies by individual centers are reporting comparable pancreas and patient survival in recipients over 50 years of age [32–34]. However, obesity still remains a significant risk factor for post-operative complications and pancreas graft loss in pancreas transplantation. A large retrospective database analysis from the USA showed that obesity was associated with a higher risk of not only post-operative complications but also pancreas graft loss, kidney graft loss, and death at 3 years [35]. Smaller, single-center studies are more optimistic, in spite of higher post-operative complications [36, 37].

It is therefore very important to thoroughly screen this high-risk group of patients before surgery and before choosing the modality of transplant.

### Simultaneous Islet-Kidney (SIK) and Islet After Kidney (IAK) Transplant

Transplantation of isolated islets of Langerhans is an accepted treatment option for patients with type 1 diabetes mellitus. In 2000, insulin independence was achieved consistently with a steroid-free immunosuppression protocol by the Edmonton group [38]. However, multiple islet transplants are required to achieve insulin independence, and long-term function remains a problem even after multiple transplants [39]. In spite of this, islet transplantation has been shown to be much safer than whole organ pancreas transplantation [40]. At present, SIK and IAK transplants are established treatment options for patients with T1DM and CKD V [41]. There are no randomized trials comparing islet transplants with whole organ pancreas transplants due to the obvious ethical reasons with regard to the different surgical procedures and the resulting complications. A retrospective study by Gerber et al. compared long-term outcomes of glucose control, renal function, and procedure-related complications between SPK and SIK transplants [40]. In terms of glucose control, SIK transplant was comparable to SPK. Endogenous insulin production by islet transplantation combined with optimal insulin therapy was shown to be sufficient to maintain near-normal glucose levels and avoid hypoglycemia. However, SPK transplantation was found to have a higher insulin independence rate (96 vs 31% in SIK group). This was at the cost of higher rate of surgical complications after an SPK transplant (40% re-laparotomies vs 0% in SIK group). Kidney function in both groups was similar. Another retrospective study by the same group compared SIK or IAK transplantation versus intensive insulin therapy (IIT) and waiting list for islet transplantation (WLI) [41]. This long-term study with more than 7-year follow-up showed that glycemic control improved significantly in the SIK/IAK group compared to the IIT/WLI groups. The rates of severe hypoglycemia also reduced significantly in the SIK/IAK group. Both these studies are limited by low patient numbers and being retrospective in nature. In spite of this, these studies demonstrate that SIK and IAK transplants are valuable alternatives to the more invasive SPK and PAK transplants. They may be suitable in those patients with significant comorbidities which preclude a whole organ pancreas transplantation and in whom better glycemic control and avoidance of severe hypoglycemias are more important than achieving insulin independence.

### Artificial Pancreas Treatment

The emerging evidence of benefits of artificial pancreas treatment in type 1 diabetes patients warrants a note about this modality of treatment. A recent systematic review and meta-analysis of 40 randomized clinical trials showed that artificial pancreas treatment was efficacious and safe in patients with T1DM [42]. The study demonstrated that the proportion of time in the near normoglycemic range (3.9–10.0 mmol/L) was significantly higher with artificial pancreas use, both overnight (weighted mean difference 15.15%, 95% confidence interval 12.21 to 18.09%) and over a 24-h period (9.62, 7.54 to 11.7%). Results were consistent in a subgroup analysis both for single hormone and dual hormone artificial pancreas systems. This has significant implications in those type 1 diabetics who have brittle diabetes and severe hypoglycemic unawareness, but who are pre-dialysis and not affected so much by the kidney failure, who may benefit the most by these artificial pancreas treatment options. However, the study noted that there were limitations of current research evidence in terms of inconsistency in outcome reporting, small sample size, and short follow-up duration of individual trials.

## Conclusions

**Fig. 1:**
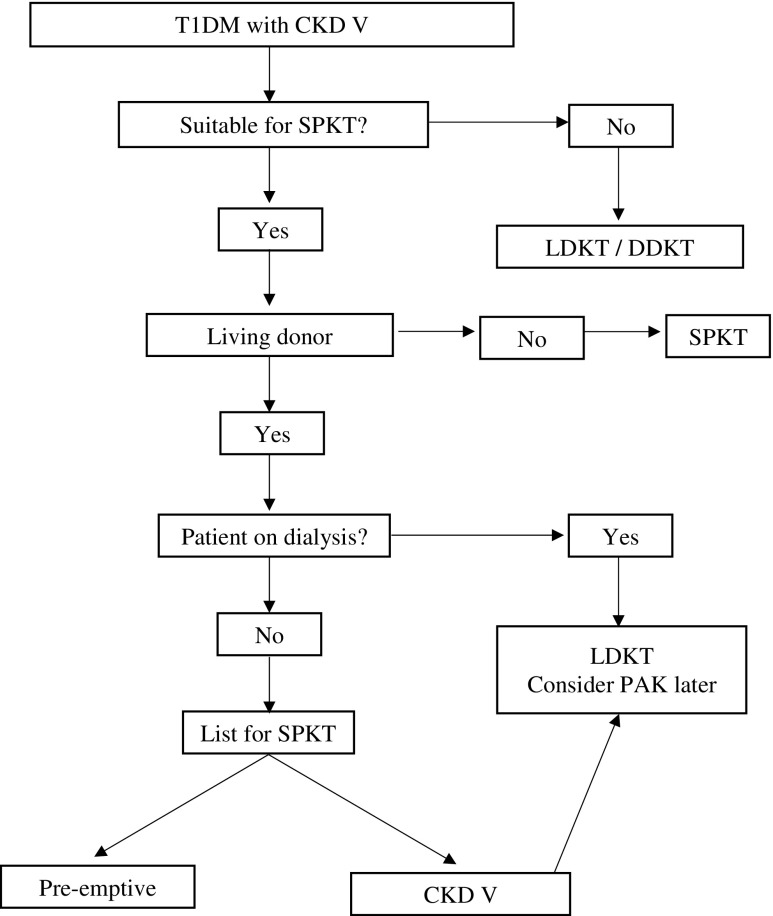
Algorithm for choosing the modality of transplant in type 1 diabetic patient with CKD V. (Adapted from Wiseman AC. Transplant Rev. (Orlando) 2013; 27:112–116, with permission from Elsevier) [43]

In summary, an SPKT or LDKT is much better than a DDKT in patients with T1DM and CKD V. The choice between an SPKT and a LDKT is more difficult and cannot be based primarily upon patient and graft survival outcomes. The modality of choice should take into consideration patients’ individual circumstances, preferences for therapy, perceived quality of life, their risk of morbidity and mortality for an SPKT, and the waiting times based on local allocation policies. Similar to the recommendations of previous such reviews [18••, 43], it is reasonable to pursue an SPKT in a pre-emptive setting. If the patient is nearing or already on dialysis, an LDKT should be considered due to the associated risk of morbidity and mortality while on the waiting list. PAK transplant is an option in these patients and should be considered within a year after LDKT. A proposed algorithm is shown in Fig. 1, reflecting the above conclusions, adapted from previous reviews [43].
